# Digital Readiness Among 3555 Individuals With Hip or Knee Osteoarthritis Initiating a Supervised Education and Exercise Therapy Programme: A Cross‐Sectional Study

**DOI:** 10.1002/msc.70127

**Published:** 2025-06-09

**Authors:** Graziella Zangger, Dorte T. Grønne, Lars H. Tang, Lau C. Thygesen, Ewa M. Roos, Søren T. Skou

**Affiliations:** ^1^ Department of Physiotherapy and Occupational Therapy The Research and Implementation Unit PROgrez Næstved‐Slagelse‐Ringsted Hospital Slagelse Denmark; ^2^ Center for Muscle and Joint Health Department of Sports Science and Clinical Biomechanics University of Southern Denmark Odense Denmark; ^3^ Department of Regional Health Research University of Southern Denmark Odense Denmark; ^4^ National Institute of Public Health University of Southern Denmark Copenhagen Denmark

**Keywords:** digital health, digital readiness, exercise therapy, health education, laten class analysis, osteoarthritis

## Abstract

**Introduction:**

Digital health can support exercise and symptom management in hip and knee osteoarthritis (OA), but uptake may depend on digital readiness (e.g., the capability) to use such tools. This study assessed digital readiness profiles in individuals with hip and/or knee OA initiating in‐person physiotherapist‐led GLA:D exercise and education and their associations with sociodemographic and health characteristics.

**Methods:**

Baseline GLA:D registry questionnaire data were analysed. The eHealth Readiness Scale measured digital readiness. Latent class analysis identified profiles, and multinomial logistic regression examined associations.

**Results:**

Among 3555 participants (mean age 66.7 years, 67% female), 53% reported confidence using the internet, 32% agreed that it improved efficiency, and only 26% agreed to use lifestyle tracking devices. Three profiles (low, intermediate, and high) were identified. Compared with the high profile, low readiness was associated with older age (odds ratio (OR) 1.96, 95% confidence interval (CI) 1.71–2.24)), female sex (OR 0.72, 95% CI 0.57–0.90), lower education (OR 0.62, 95% CI 0.45–0.88), living alone (OR 1.39, 95% CI 1.11–1.76), and more comorbidities (OR 1.10, 95% CI 1.04–1.17). The intermediate profile showed similar trends but were also associated with less obesity (0.75, 95% CI 0.60–0.95) and lower walking speed (0.72, 95% CI 0.53–0.97).

**Conclusions:**

Digital readiness profiles differed notably by age, sex, and education, underscoring the importance of readiness to enhance uptake and guide implementation and resource allocation of digital health in OA care. Future studies should address digital readiness improvement strategies.

## 1 Background

The high and increasing prevalence of hip and knee osteoarthritis (OA) (Steinmetz et al. 2023) highlights the importance of innovative approaches to disease management, including the integration of digital health solutions. Telehealth platforms, smartphone applications (apps), and wearable devices can provide continuous personalised support and care, monitor symptoms, and facilitate remote communication, which is essential for managing chronic conditions such as hip and knee OA (Shah et al. 2022). Digital solutions further offer benefits such as reduced travel, shorter waiting times, lower costs, greater flexibility, and intensified and practical care (World Health Organization 2021). Adapting to digital health solutions is becoming increasingly important in healthcare (World Health Organization 2019), and embracing digital health solutions in hip or knee OA management represents a proactive approach to mitigating the long‐term impacts of OA on both the individual and healthcare systems (Fernandes et al. 2013; Hunter and Bierma‐Zeinstra 2019; Nelson et al. 2014).

Digitally delivered exercise programs are particularly intriguing as they offer an alternative to traditional rehabilitation for individuals with hip and knee OA (Bunting et al. 2021; De Santis et al. 2022), especially for those with severe functional limitations or commitments that hinder in‐person participation (World Health Organization 2021; De Santis et al. 2022; Merolli et al. 2022). However, common barriers to usage include privacy concerns, reduced personal interaction, and doubts about the credibility of digital solutions (World Health Organization 2021). In addition, a persistent digital divide affects older adults, individuals with low digital (health) literacy, and those from socioeconomically disadvantaged backgrounds, who may face challenges in accessing and using digital technologies effectively (World Health Organization 2021; Arias López et al., 2023; Zangger et al. 2024). These disparities highlight that not all are equally ready to engage with digital health solutions.

While existing studies have demonstrated several health benefits of digitally delivered exercise for individuals with hip or knee OA (Hinman et al. 2024; McHugh et al. 2022; Nelligan, Hinman, Kasza, et al. 2021; Yang et al. 2023; Zangger et al. 2023), a knowledge gap remains in understanding how ready individuals with hip or knee OA are to start engaging with these digital health solutions (Horrigan 2016). This can be determined by measuring digital readiness, which refers to the combination of motivation, willingness, confidence, and capability needed to initially accept and engage with digital health solutions (Blut and Wang 2020; Yusif et al. 2017). Various frameworks have explored readiness to use digital health, suggesting that diverse personal, social, and contextual factors influence digital readiness (Kayser et al. 2019; Phillips et al. 2018; Rising et al. 2024; Scherrenberg et al. 2023). While closely related to digital health literacy—the ability to find, understand, and use health information via technology (Norman and Skinner 2006)—digital readiness may extend beyond this by incorporating emotional and social dimensions of use (Rising et al. 2024). Using the eHealth Readiness Scale (Bhalla et al. 2016) level to assess digital readiness, self‐efficacy (a person's confidence in the ability to use digital solutions) is emphasised as a central component. The eHealth Readiness scale provides a low‐burden measurement tool that accounts for both the psychological and practical aspects of engaging with digital health solutions (Bhalla et al. 2016).

Understanding digital readiness is essential for optimising the implementation and uptake of digital health interventions, as users must go beyond the first access to meaningfully engage with these solutions to succeed. Furthermore, identifying and stratifying individuals based on digital readiness and related factors may enable more tailored and inclusive intervention strategies, ensuring that digital health interventions are accessible, effective, and aligned with user needs. Digital readiness can therefore be a pivotal aspect of maximising potential benefits and serves as a foundation for the successful design, implementation, and sustainability of digital health solutions (Kavandi and Jaana 2020; Martin 2012; Rising et al. 2024; Whitten et al. 2010). Therefore, this study aims to investigate digital readiness profiles among people with hip or knee OA initiating in‐person physiotherapist‐supervised exercise therapy and education in primary care (GLA:D) and assess associations with sociodemographic and health characteristics and performance‐based functional test outcomes with the established digital readiness profiles.

## 2 Methods

The study follows the Strengthening the Reporting of Observational Studies in Epidemiology (STROBE) guideline (von Elm et al. 2007). The STROBE checklist can be found in Supporting Information S1: Table 1. The statistical analysis plan was preregistered before analysis and is available at www.osf.io/sbpw4.

### 2.1 Study Design and Data Sources

This cross‐sectional study used national GLA:D registry data. GLA:D offers guideline‐aligned group education and supervised exercise therapy for people with hip or knee OA (Skou and Roos 2017) through 2–3 90‐min education sessions and 12 60‐min supervised neuromuscular exercise sessions delivered twice weekly for six weeks. Approximately 6000 participants initiate GLA:D annually through self‐referral or referral from a general practitioner at one of the nearly 300 Danish physiotherapy clinics, either private (with ∼40% public reimbursement) or public (free attendance).

The GLA:D registry collects data from therapists and participants at baseline, 3 months, and 12 months after baseline; only baseline data are used in this study. Participants received an emailed link to an online questionnaire with up to two reminders after 1 week, and onsite kiosk options were available for participants without email.

### 2.2 Participants

Participants who initiated GLA:D between 7 March 2022, and 5 January 2023, were included, as digital readiness information was collected during this period. The inclusion criterion in GLA:D is a clinical diagnosis of hip or knee OA. The exclusion criteria are reasons other than OA for joint problems (e.g., tumour, inflammatory joint disease, or sequelae after a hip fracture), having other competing and more severe symptoms than OA problems (e.g., chronic, generalised pain, or fibromyalgia), and being unable to read and understand Danish. Radiographic images are not needed for a clinical diagnosis of OA (Sakellariou et al. 2017) and are therefore not a criterion for entering GLA:D.

### 2.3 Variables of Interest

#### 2.3.1 Digital Readiness

The eHealth Readiness Scale was developed in 2016 by Bhalla et al. to measure participants' readiness to engage in eHealth or digital health interventions (digital readiness) (Bhalla et al. 2016). The scale items are based on Bandura's theory on self‐efficacy, previous literature, and measurement scales (Bhalla et al. 2016). The seven‐item scale is scored on a six‐point Likert‐type scale (1 = strongly disagree and 6 = strongly agree), and scores range from 7 to 42, with higher scores indicating greater readiness with no presiding thresholds of (in)sufficient readiness (Bhalla et al. 2016). The scale has previously demonstrated good psychometric properties with a robust unidimensional scale and high internal consistency (Cronbach's alpha 0.81) (Bhalla et al. 2016). While Bhalla et al. assessed digital readiness in the continuation of a digital health intervention (Bhalla et al. 2016), we assessed baseline readiness without referencing a specific digital solution or measuring uptake in this study.

##### 2.3.1.1 Linguistic and Cross‐Cultural Validation

The eHealth Readiness Scale, including the introduction text, was independently forward and backward translated from English to Danish by two health professionals (one native Danish speaker fluent in English and one native English speaker fluent in Danish). Dr. Bhalla approved the use and translation of the scale. Two patient‐partners reviewed and commented on the Danish translation. A third researcher resolved discrepancies by incorporating patient feedback to finalise the Danish version (Supporting Information S1: Table 2).

##### 2.3.1.2 Digital Readiness Profiles

As the eHealth Readiness Scale has no approved cutoff for readiness levels, an explorative approach was chosen using latent class analysis to identify subgroups on the basis of the participant responses to the scale items.

#### 2.3.2 Sociodemographic Characteristics

Age and sex were derived from the Danish Civil Registration (CPR) System (Pedersen 2011), with age continuously calculated from the initial visit date. Sex was recorded as male/female. We recorded whether participants were born in Denmark and had Danish citizenship as binary variables (yes/no). Birthplace and citizenship were included as proxies for language proficiency and cultural integration. Education level was based on the highest level completed and categorised into no, primary, or lower secondary (collapsed), upper secondary, and higher education. Cohabitation status was recorded, indicating whether the participant lived alone or with others.

#### 2.3.3 Health Characteristics

Body mass index (BMI) was calculated on the basis of body weight in kilograms and height in centimetres (measured by the therapist), which was subsequently categorised into < 18.4 to 24.9 for underweight and normal weight, 25.0 to 29.9 for preobese, and ≥ 30 for obese classes I to III (‘Obesity,’ 2000). The participants indicated the most affected (symptomatic) hip or knee joint. Therapists recorded the duration of symptoms in months for the most affected joint. Pain intensity (of the most affected joint) during the last week was measured via the visual analogue scale (VAS) (Hawker et al. 2011), which ranges from 0 to 100 mm (indicating 'no pain' to 'maximum pain'). Therapists recorded analgesic use, including acetaminophen, nonsteroidal anti‐inflammatory drugs (NSAIDs), and opioid medication, over the preceding 2 weeks. Comorbidities were assessed by quantifying the number of self‐reported health conditions from 30 possibilities.

Moderate‐to‐vigorous physical activity (MVPA) and vigorous physical activity (VPA) were based on self‐reported time spent engaging in either intensity on a typical week (MVPA: < 30 min, 30–89 min, 90–149 min, 150–299 min, or > 300 min; VPA: < 30 min, 30–59 min, 60–89 min, 90–149 min, or > 150 min). Physical activity compliance was determined in a binary manner, adhering to the WHO minimum recommendations for adult physical activity (≥ 150 min of moderate physical activity, ≥ 75 min of VPA, or an equivalent combination). This calculation was similar to the Nordic Physical Activity Questionnaire‐short validation method (Danquah et al. 2018). Sedentary behaviour was calculated by typically daily sitting time during transportation, work/school, leisure/screen time or other activities, categorised with a threshold of ≥ 9 h per day indicating sedentary behaviour (Ku et al. 2018). Values > 16 h for work/school, > 6 h for other sedentary behaviours, or > 24 h were excluded.

The summary scores of the Hip disability and Osteoarthritis Outcome Score 12 (HOOS‐12) and the Knee disability and Osteoarthritis Outcome Score 12 (KOOS‐12) questionnaires were utilised to assess pain, function, and quality of life. Scores are given on a scale of 0–100 (higher scores indicate better quality of life) (Gandek et al. 2019a, 2019b). The EuroQol 5‐Dimensions (EQ‐5D) 5‐Level assesses health‐related quality of life across five dimensions with five response levels in an index score. The index scores were based on the Danish value set (Jensen et al. 2021). Furthermore, the EQ‐5D VAS scores overall self‐rated health from 0 to 100 (higher scores indicate better quality of life) (Jensen et al. 2021).

#### 2.3.4 Functional Performance‐Based Tests

Functional performance was assessed using the 30‐s chair‐stand test and the 40‐m fast‐paced walk test, as recommended by the Osteoarthritis Research Society International (OARSI) (Dobson et al. 2013). Both tests were administered once at the respective clinics according to OARSI protocols (Dobson et al. 2013). The chair‐stand test recorded the number of completed stands in 30 s, while the walk test measured walking speed (m/s) over 40 m. The use of walking aids during testing was documented.

### 2.4 Nonresponders

A nonresponder analysis was performed for the available items: age, sex, BMI, most affected joint, analgesic use, results from the 30‐s chair‐stand test and the 40‐m fast‐paced walk test, as well as available email contact.

### 2.5 Statistical Analysis

Descriptive statistics were computed to summarise participants' sociodemographic and health characteristics, performance‐based test results, and responses to the eHealth Readiness Scale items. Categorical data are presented as absolute frequencies and percentages, whereas continuous data are expressed as the means with standard deviations (SDs) or medians with interquartile ranges (IQRs) as appropriate. Scale reliability was tested via Cronbach's alpha, inter item, and item‐rest tests for internal consistency.

Latent class analysis was performed using Mplus 8.10 (Muthén and Muthén 1998). Latent class analysis is a probabilistic model‐based technique used to categorise a sample into distinct and exhaustive subgroups according to their response pattern to the eHealth Readiness Scale (Kongsted and Nielsen 2017). Models with one to five classes were assessed, including models where the response categories were collapsed for similar answer categories (i.e., strongly agree with agree, mildly agree with mildly disagree, and strongly disagree with disagree). The final number of classes was determined on the basis of conceptual meaning, subgroup size, and entropy statistics, indicating the certainty of subgroup membership (Nylund et al. 2007). Maximum likelihood estimation via the expectation‐maximisation procedure was employed with automatically generated random starting values and 1000 iterations to enhance generalisability. Entropy statistics were evaluated at a threshold of ≥ 0.80 to ascertain the optimal number of latent classes.

Multinomial logistic regressions followed class determination and examined associations between digital readiness profiles (categorical outcome) and selected sociodemographic, health, and performance‐based variables (exposures) in a single model. One readiness profile was set as the base reference. Results are reported as odds ratios (ORs) with 95% confidence intervals (CIs). Age was rescaled by 10 to reflect a decade‐level increase; VAS pain, KOOS‐12/HOOS‐12, and EQ‐5D VAS scores were similarly rescaled to reflect a 10‐point decrease. Only the EQ‐5D VAS was included in the multinomial logistic regressions to avoid multicollinearity. Multicollinearity was assessed via variance inflation factors (VIF) from linear regression using the same predictors. All VIFs were below 3.1; see Supporting Information S1: Table 3.

Potential confounders were assessed by adding age, sex, BMI, education, and sedentary behaviour stepwise to unadjusted multinomial logistic models to examine changes in odd ratios. Interaction effects between age, sex, BMI, and education were tested using models with interaction terms to assess whether associations with digital readiness profiles varied by combined characteristics.

The data were analysed via STATA version 18 (StataCorp. Stata Statistical Software: Release, n.d., 18 April 2023, College Station, TX: StataCorp LLC. n.d.) at a significance level of *p* < 0.05 (two‐tailed).

## 3 Results

### 3.1 Participant Characteristics

A total of 3555 out of 4776 participants responded to the survey (74%) and were included in the study. The participants were primarily older adults (mean age 66.4 years, SD 9.6), with a majority being female (67%) and the knee being the most affected joint (Table 1).

**TABLE 1:** Sociodemographic and health characteristics and performance‐based functional tests of the total group and the three digital readiness profiles.

Variable	All	Low digital readiness profile	Intermediate digital readiness profile	High digital readiness profile
Participants, *n* (%)	3555	740 (20.8%)	1528 (43.0%)	1287 (36.2%)
Age at first visit, mean (SD)	66.4 (9.6)	70.4 (8.9)	66.7 (9.4)	63.8 (9.4)
Female, *n* (%)	2386 (67.1%)	514 (69.5%)	1083 (70.9%)	789 (61.3%)
Born in Denmark, *n* (%)	3414 (96.0%)	716 (96.8%)	1470 (96.2%)	1228 (95.4%)
Danish citizenship, *n* (%)	3500 (98.5%)	733 (99.1%)	1509 (98.8%)	1258 (97.7%)
Education level, *n* (%)				
No, primary, or lower secondary education	376 (10.6%)	128 (17.3%)	164 (10.7%)	84 (6.5%)
Upper secondary education	1675 (47.1%)	360 (48.6%)	761 (49.8%)	554 (43.0%)
Higher education	1504 (42.3%)	252 (34.1%)	603 (39.5%)	649 (50.4%)
Cohabitate status, living alone, *n* (%)	945 (26.6%)	248 (33.5%)	417 (27.3%)	280 (21.8%)
BMI, mean (SD)	28.7 (5.5)	28.3 (5.4)	28.6 (5.6)	29.0 (5.3)
BMI category, *n* (%)^a^				
Underweight/normal weight	942 (26.5%)	218 (29.5%)	420 (27.5%)	304 (23.6%)
Preobese	1351 (38.0%)	278 (37.6%)	581 (38.0%)	492 (38.2%)
Obese (class I, II, or III)	1215 (34.2%)	238 (32.2%)	509 (33.3%)	468 (36.4%)
Self‐reported most affected joint, *n* (%)				
Knee	2362 (66.4%)	485 (65.5%)	1022 (66.9%)	855 (66.4%)
Hip	1193 (33.6%)	255 (34.5%)	506 (33.1%)	432 (33.6%)
Bilateral symptoms, *n* (%)	1350 (38.0%)	263 (35.5%)	555 (36.3%)	532 (41.3%)
Symptoms length of most affected joint in months, median (IQR)	12.0 (6.0–27.0)	12.0 (6.0–30.0)	12.0 (5.0–24.0)	12.0 (5.0–30.0)
Hip or knee pain during the last week (0–100), mean (SD)	47.0 (22.8)	48.3 (23.8)	47.1 (22.3)	46.0 (22.8)
Taking pain medications, *n* (%)	2277 (64.1%)	487 (65.8%)	988 (64.7%)	802 (62.3%)
Number of comorbidities, mean (SD)	2.4 (1.9)	2.9 (2.0)	2.4 (1.9)	2.2 (1.8)
Three most common comorbidities, *n* (%)^b^				
Hypertension	1525 (42.9%)	382 (51.6%)	637 (41.7%)	506 (39.3%)
Back pain	1092 (30.8%)	259 (35.0%)	470 (30.8%)	363 (28.2%)
Hypercholesterolaemia	1083 (30.5%)	268 (36.2%)	466 (30.5%)	349 (27.1%)
Moderate to vigorous physical activity, *n* (%)				
< 30 min	783 (22.0%)	335 (21.9%)	187 (25.3%)	261 (20.3%)
30–89 min	1024 (28.8%)	445 (29.1%)	219 (29.6%)	360 (28.0%)
90–149 min	688 (19.4%)	302 (19.8%)	112 (15.1%)	274 (21.3%)
150–299 min	724 (20.4%)	318 (20.8%)	142 (19.2%)	264 (20.5%)
> 300 min	336 (9.5%)	128 (8.4%)	80 (10.8%)	128 (9.9%)
Noncompliant with WHO's minimum recommendations for physical activity, *n* (%)	2389 (67.2%)	496 (67.0%)	1044 (68.3%)	849 (66.0%)
Sedentary behaviour (sitting > 9 h/day), *n* (%)^c^	1633 (45.9%)	235 (31.9%)	607 (39.8%)	595 (46.4%)
eHealth readiness scale score, range 7–42, mean (SD)	25.9 (8.1)	14.0 (4.1)	25.3 (3.4)	33.7 (3.9)
KOOS/HOOS 12 summary score, mean (SD)	51.5 (14.6)	51.0 (15.7)	51.0 (14.4)	52.1 (14.9)
EQ‐5D index, mean (SD)	0.758 (0.202)	0.743 (0.218)	0.754 (0.202)	0.772 (0.192)
EQ‐5D VAS, mean (SD)	67.4 (19.2)	65.1 (20.9)	67.3 (18.8)	68.7 (18.7)
Number of chair stands (30 s chair stand test), mean (SD)	11.9 (4.2)	11.1 (3.9)	11.8 (4.1)	12.5 (4.3)
Walking speed in metres per second (40 m walk test), mean (SD)	1.47 (0.35)	1.38 (0.36)	1.45 (0.33)	1.55 (0.35)

Abbreviations: BMI = Body Mass Index, EQ‐5D = EuroQol 5‐Dimension 5‐Level, HOOS = Hip disability and Osteoarthritis Outcome Score, IQR = Interquartile range, KOOS = Knee injury and Osteoarthritis Outcome Score, SD = Standard deviation, VAS = Visual Analogue Scale, WHO = World Health Organisation.

^a^ *n* = 47 participants had missing BMI data.

^b^ Differences in the prevalence of the three most common comorbidities across digital readiness profiles were assessed by chi‐square test (*χ* ^2^) and found significant (*p* ≤ 0.007).

^c^ *n* = 11 participants were excluded from the analysis due to missing data or sedentary time limits.

### 3.2 Nonresponders

The 1221 nonresponders were older (mean age 67.3 years, SD 11.0) and more often lacked an email contact (8.7%). The nonresponders also had the knee as the most symptomatic joint but performed worse on the functional performance‐based tests than the responders (Supporting Information S1: Table 4).

### 3.3 Digital Readiness

#### 3.3.1 Reliability of the eHealth Readiness Scale

The Cronbach's alpha of the eHealth Readiness Scale was 0.90, with high item‒test scores (range 0.72–0.85) and average inter item covariance (1.20) (Supporting Information S1: Tables 5 and 6).

#### 3.3.2 eHealth Readiness Responses

**FIGURE 1:**
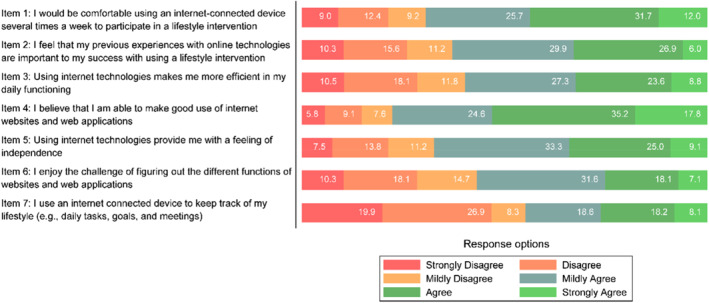
The eHealth readiness scale item response distribution in percentage.

The mean eHealth Readiness Scale score was 25.9 (SD 8.1). More than half of the participants (53.0%) agreed or strongly agreed that they could make good use of the internet, web apps, or apps (item 4), whereas 21.4% disagreed or strongly disagreed. However, only 32.4% agreed or strongly agreed that internet technologies made them more efficient (item 3), and 26.3% of the participants agreed or strongly agreed to use an internet‐connected device to track their lifestyle (item 7), whereas 46.8% disagreed or strongly disagreed (Figure 1).

### 3.4 Digital Readiness Profiles

**FIGURE 2:**
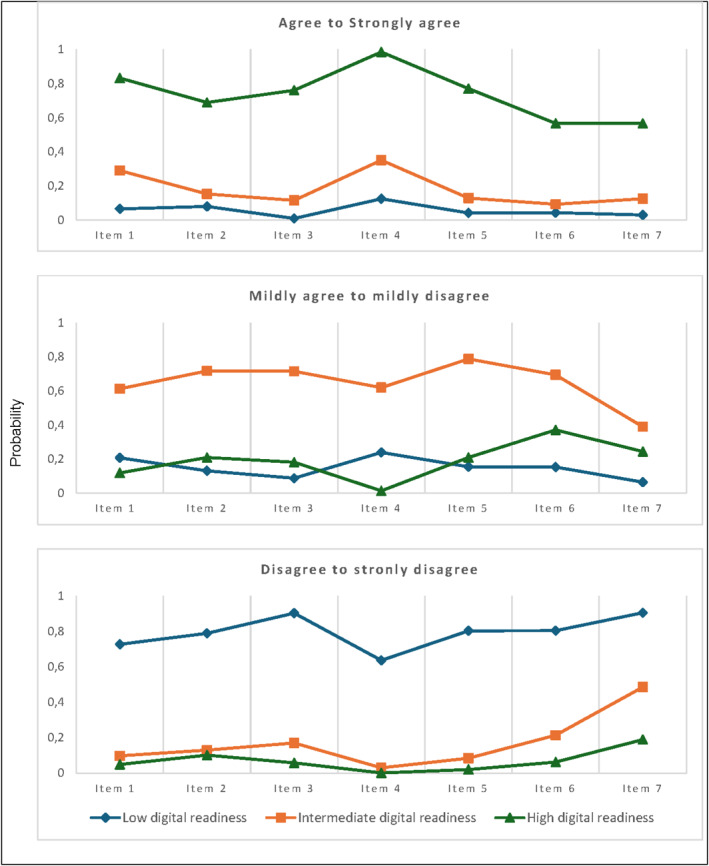
The latent class analysis of the 3‐class model with the probabilities of the profiles within the collapsed response categories.

Five latent class analysis models were assessed. An even participant distribution within the 3‐, 4‐, and 5‐class models (Supporting Information S1: Tables 7–10) was found, and all had a good statistical fit (entropy ≥ 0.87). Consequently, we opted for a simplified model comprising the three classes with collapsed response categories, as this model aligned well with the interpretability and clinical meaningfulness of the eHealth Readiness Scale. The 3‐class model showed more distinct digital readiness profiles than the 4‐ and 5‐class models, and the mean probabilities for class membership were high (0.94 for class 1 (low digital readiness), 0.94 for class 2 (intermediate digital readiness), and 0.96 for class 3 (high digital readiness)) (Figure 2).

The participants with low digital readiness were older and predominantly female, whereas those with high readiness had higher BMIs, more bilateral symptoms, less pain, and fewer comorbidities. Hypertension, back pain, and hypercholesterolaemia were the most prevalent comorbidities, which differed significantly by readiness profile. Despite slightly greater sedentary behaviour, the high‐readiness profile had better quality of life and performance‐based test results (Table 1).

#### 3.4.1 Factors Associated With the Digital Readiness Profiles

Older individuals showed significantly lower digital readiness, whereas higher education corresponded to higher readiness. Men were more often in the high readiness profile than women. Living alone and having lower self‐reported quality of life were more common in the low‐readiness profile than in the high‐readiness profile. A greater number of comorbidities were observed in the low profile than in both the intermediate and high profiles. Sedentary behaviour was associated with higher readiness. Functional performance tests had limited influence, although a higher walking speed was less likely in the intermediate profile than in the high profile. See Table 2.

**TABLE 2:** Multinomial logistic regression analyses of the digital readiness profiles and sociodemographic characteristics, health outcomes, and functional performance‐based test outcomes.

	Low digital readiness profile vs. high (ref. High digital readiness profile)		Intermediate digital readiness profile vs. high (ref. High digital readiness profile)		Low digital readiness profile vs. intermediate (ref. intermediate digital readiness profile)	
Variable	Or (95%CI)	*p* value^a^	Or (95% CI)	*p* value^a^	Or (95% CI)	*p* value^a^
Age at first visit (per 10‐year increase)	1.96 (1.71–2.24)	< 0.001	1.30 (1.18–1.44)	< 0.001	1.50 (1.32–1.71)	< 0.001
Sex, male (ref. Female)	0.72 (0.57–0.90)	0.004	0.65 (0.55–0.78)	< 0.001	1.10 (0.89–1.36)	0.381
Born in Denmark (ref. yes)	0.85 (0.43–1.65)	0.632	1.12 (0.69–1.82)	0.649	0.76 (0.40–1.42)	0.388
Danish citizenship (ref. yes)	0.59 (0.20–1.73)	0.338	0.58 (0.27–1.24)	0.160	1.02 (0.35–2.95)	0.975
Education level (ref. no, primary, or lower secondary education)		< 0.001		< 0.001		0.004
Upper secondary education	0.62 (0.45–0.88)		0.86 (0.63–1.16)		0.73 (0.55–0.98)	
Higher education	0.33 (0.23–0.47)		0.55 (0.40–0.75)		0.60 (0.44–0.81)	
Cohabitate status, living alone (ref. living with others)	1.39 (1.11–1.76)	0.005	1. 13 (0.93–1.37)	0.211	1.23 (1.00–1.52)	0.052
BMI category (ref. Underweight/normal weight)^b^		0.090		0.047		0.806
Preobese	0.83 (0.64–1.07)		0.90 (0.73–1.11)		0.92 (0.73–1.17)	
Obese (class I, II, or III)	0.73 (0.54–0.97)		0.75 (0.60–0.95)		0.96 (0.74–1.26)	
Self‐reported most affected joint, hip (ref. Knee)	0.98 (0.78–1.22)	0.848	0.96 (0.80–1.14)	0.619	1.02 (0.83–1.26)	0.828
Bilateral symptoms (ref. no)	0.91 (0.73–1.13)	0.388	0.92 (0.77–1.09)	0.319	0.99 (0.81–1.22)	0.935
Symptoms length of most affected joint in months	1.00 (1.00–1.00)	0.313	1.00 (1.00–1.00)	0.607	1.00 (1.00–1.00)	0.527
Hip or knee pain during the last week (per 10‐point increase)	0.96 (0.90–1.02)	0.214	0.98 (0.93–1.03)	0.371	0.98 (0.93–1.04)	0.568
Taking pain medications (ref. no)	0.88 (0.70–1.11)	0.288	0.96 (0.80–1.15)	0.678	0.92 (0.74–1.14)	0.253
Number of comorbidities	1.10 (1.04–1.17)	0.001	1.01 (0.96–1.06)	0.676	1.09 (1.02–1.15)	0.002
Compliant with WHO's minimum recommendations for physical activity (ref. Noncompliant)	1.11 (0.89–1.38)	0.345	0.99 (0.82–1.17)	0.868	1.13 (0.92–1.38)	0.713
Nonsedentary behaviour (ref. Sedentary (sitting > 9 h/day)^c^	1.63 (1.32–2.02)	< 0.001	1.21 (1.02–1.42)	0.024	1.35 (1.10–1.65)	0.019
KOOS 12/HOOS 12 summary score (per 10‐point increase)	0.96 (0.87–1.07)	0.463	0.98 (0.90–1.06)	0.611	0.98 (0.89–1.08)	0.722
EQ‐5D VAS (per 10‐point increase)	0.92 (0.87–0.98)	0.011	0.97 (0.92–1.03)	0.283	0.95 (0.90–1.00)	0.072
Number of stands (30‐s chair stand test)	0.97 (0.94–1.00)	0.047	0.99 (0.96–1.01)	0.245	0.98 (0.94–1.01)	0.251
Walking speed metres per second (40‐m walk test)	0.79 (0.53–1.16)	0.228	0.72 (0.53–0.97)	0.031	1.10 (0.76–1.60)	0.623

Abbreviations: BMI = Body Mass Index, CI = Confidence Interval, EQ‐5D = EuroQol‐5 Dimension, HOOS = Hip disability and Osteoarthritis Outcome Score, KOOS = Knee injury and Osteoarthritis Outcome Score, OR = Odds Ratio, Ref. = Reference category, SD = Standard Deviation, VAS = Visual Analogue Scale, WHO = World Health Organisation.

^a^ *p* value of a type‐3 test for the overall effect.

^b^ *n* = 47 participants had missing BMI data.

^c^ *n* = 11 participants were excluded from the analysis due to missing data or sedentary time limits.

Confounder analyses indicated that sex and BMI modestly influenced the associations, but the interactions were nonsignificant (Supporting Information S1: Tables 11–14). In contrast, higher education emerged as a confounder as it attenuates the age effect on digital readiness and substantially reduces the likelihood of belonging to low or intermediate digital readiness groups compared with high (Supporting Information S1: Tables 14–17).

## 4 Discussion

This study is the first to assess digital readiness in individuals with hip or knee OA, revealing sociodemographic and health‐related factors associated with digital readiness levels. On average, the participants had moderate digital readiness scores that varied widely. The participants had moderate and widely varying scores, with approximately half agreeing to be proficient in internet and app use, yet only a quarter tracking their lifestyle digitally and a third acknowledging increased efficiency from internet technologies. We identified three distinct digital readiness profiles: low, intermediate, and high, differing by age, sex, educational level, number of comorbidities, cohabitation status, overall self‐reported health, sedentary behaviour, and walking speed, with older age and lower education being more prominent factors for low readiness. These findings suggest that factors that enhance digital readiness and address digital disparities in OA care should be considered in optimising the uptake and use of digital health interventions in clinical practice.

The apparent low utilization rate of digital devices for lifestyle tracking in the study population could indicate lower digital readiness or be specifically related to devices or apps. This should be further investigated as we did not measure the actual uptake of digital solutions. However, a study that assessed eHealth readiness among 2602 older adults reported that while more than half of the participants could find health information online, very few used health‐related apps (Gordon and Hornbrook 2018). This could be due to known barriers (World Health Organization 2021). However, many existing apps simply lack the quality to facilitate effective behaviour change, particularly in OA (Bricca et al. 2022). Additionally, language barriers and financial constraints imposed by paywalls may further impede the use of apps for tracking and managing lifestyle behaviours in this population (Bricca et al. 2022). This calls for improved design to enhance app engagement in OA management.

Although few studies have specifically examined digital readiness profiles, some of our findings align with existing literature. In a study on individuals with cancer, age, educational attainment, cohabitation status, number of comorbidities, and physical activity levels varied significantly across digital readiness profiles (Rossen et al. 2019). Those with lower readiness were typically older, less educated, living alone, and managing multiple chronic conditions, consistent with our own results. A Norwegian study of older adults receiving home care identified the least digitally ready group as older, less educated, and with minimal access to digital tools (Bergh et al. 2025). Although some participants showed potential to benefit from digital health solutions, many required significant support or preferred non‐digital alternatives (Bergh et al. 2025).

In contrast, a study of people with diabetes identified no clear differences in sociodemographic or health characteristics across profiles. However, specific subgroups, such as younger individuals with mental health challenges and older adults with limited digital experience, were identified as having notably lower digital readiness (Thorsen et al. 2020). Interestingly, a study of patients with implantable cardioverter‐defibrillators reported lower readiness among younger individuals (Rosenmeier et al. 2025), which suggests that the nature of the health condition may influence digital engagement in different ways. These findings support our findings that age and education level are key determinants of digital readiness, though the influence of condition‐specific factors should not be overlooked.

Other studies exploring digital readiness in chronic conditions have relied on study‐specific measures or assessed readiness based on general internet access and usage rather than identifying readiness profiles. For instance, a large general population and diabetes sample assessed readiness using a single Likert‐scale item, ‘I am not ready for eHealth,’ and found that nearly half of the 2895 participants reported low readiness, with even higher proportions (76%) among people with diabetes [426]. In a heart failure population, readiness to use the internet was assessed using a transtheoretical model‐based staging tool using study‐specific readiness items. They reported that only 23% were active online users, yet 44% of non‐users expressed willingness to adopt eHealth with proper access and support, highlighting the role of external barriers rather than intrinsic resistance (Treskes et al. 2019). However, these studies focused on readiness for digital medical management and not targeting lifestyle changes.

Broader investigations into digital inequalities have similarly identified key factors, such as age, education, and socioeconomic status, as strong predictors of access and engagement with digital tools. For example, a study across 28 European member states found that these factors influenced the use of e‐services, mobile apps, and social networks, reinforcing the relevance of our findings in a wider digital health context (Elena‐Bucea et al. 2021). For age, they reported that younger generations were more digitally engaged, particularly with social networks, with age as the primary factor for inequality. Additionally, Gorden et al. reported that although most seniors could access the internet from home, either independently or with assistance, this ability was significantly lower with each 5‐year increase in age (Gordon and Hornbrook 2018). Similarly, we observed that older individuals were likely to exhibit low digital readiness.

For educational attainment, Elena‐Bucea et al. also reported that higher education increased e‐service use (Elena‐Bucea et al. 2021). Our results supported this finding, with a strong gradient across all three profiles, with higher education associated with greater digital readiness. However, only the highest education level significantly differed between the intermediate and high profiles. This suggests that although there may be a relationship between education and digital readiness, this could indicate a segment of the OA population with some digital engagement but insufficient skills or confidence to be highly digitally ready.

In our study, males were more often in the high‐readiness profile than in the low‐readiness profile. However, there was no significant difference between the low‐ and intermediate‐readiness profiles. Elena‐Buce et al. reported minimal differences between the sexes in e‐services and social network use, but males tended to use them more (Elena‐Bucea et al. 2021). Differences in preferences for or access to digital tools between the sexes may exist.

Intriguingly, our results also revealed that obesity and sedentary behaviour were associated with high digital readiness, whereas walking speed increased the likelihood of being in the high profile. Other studies have pointed to a relationship between higher education and prolonged sitting and reported that higher education was associated with more total sitting time and less nonwork sitting, possibly due to greater leisure‐time physical activity engagement (Hadgraft et al. 2015; Piirtola et al. 2020). This may be due to the population being resourceful despite having a higher BMI and sedentary behaviour. A higher BMI in the high‐readiness profile may also reflect differences in the proportions of males and females. Although we found no signs of interaction on the basis of sex, we cannot rule out confounding factors. Our findings may reflect a chance finding or unidentified factors, and further research is needed to investigate the underlying mechanisms involved.

Our findings showed that age, sex, and education significantly influence higher digital readiness levels, a pattern that likely extends beyond individuals with OA and applies broadly across different populations. Our study suggests that having more chronic conditions is associated with low and intermediate digital readiness. This is particularly relevant for older adults who are more susceptible to developing chronic conditions. Managing multiple chronic conditions often involves complex healthcare needs, which may amplify the challenges of using digital health for lifestyle changes (Skou et al. 2022; Smith et al. 2021).

Social support has been highlighted as a key facilitator of digital use, and studies have shown that support can positively shape attitudes and behaviours towards technology (Kebede et al. 2022; Knapova et al. 2020). This may shed light on our finding that living alone increases the likelihood of being in the low profile, but it does not explain why we did not find the same for the intermediate versus the high profile.

Barriers such as motivation, attitudes, physical limitations, cognitive ability, and technological knowledge and skills have been identified for digital nonuse and initial adoption among older adults (Kebede et al. 2022). To some extent, these barriers align with our results, as declines in physical and cognitive abilities are often linked to age and comorbidity burden, and technological skills could be related to education level. Therefore, the number of chronic conditions should be considered when designing and implementing digital health interventions, which could impact an individual's ability to adopt and effectively use these solutions, especially considering that age and education level are essential for high digital readiness and, hence, the uptake of the digital health solution. Notably, Nelligan et al. reported that comorbidities did not moderate outcomes in a digital hip and knee OA exercise programme (Nelligan et al. 2021). This suggests equal benefits if barriers to the use of digital health solutions are addressed and if the solutions are tailored to specific needs.

### 4.1 Strengths and Limitations

A strength of this study is that we included 3555 individuals, which provides high power for the latent class analysis, capturing digital readiness profiles and related characteristics of individuals with hip or knee OA motivated to change their lifestyle. This real‐world clinical relevance provides insights into digital readiness in OA management; however, selecting individuals from an exercise therapy and education programme in a high‐income country with high technology and healthcare access may limit its generalisability to a broader population.

The cross‐sectional design restricts causality, and the reliance on self‐reported data introduces potential recall and response biases. Potential selection bias also exists due to the online collection of questionnaires, as participants may be more digitally competent. Physical activity was measured categorically, limiting precision, and sedentary behaviour was simplified by truncating high values, which may obscure subtle variations. Future studies should use objective measures for greater accuracy. Despite not undergoing a complete psychometric evaluation, the eHealth Readiness Scale demonstrated high reliability, and we improved the translation with patient‐partner feedback. Furthermore, we examined digital readiness profiles as a foundation for understanding digital adoption, but we did not evaluate whether digital readiness is associated with actual uptake. Future research should assess whether digital readiness, as defined by the eHealth Readiness Scale, directly correlates with the adaptation and utilization of digital solutions. Subjectivity in interpreting latent class analysis results may have led to the identification of a suboptimal number of classes, which will require validation in an independent sample. Birthplace and citizenship may not fully capture sociodemographic disparities. Future studies should assess digital readiness across diverse populations and settings by incorporating more accurate sociodemographic and socioeconomic factors. We assessed confounders and interaction, and although we found only limited confounding and interaction, we cannot exclude the influence on the results.

### 4.2 Implication

Our findings are relevant for individuals with hip or knee OA, as effective self‐management and adherence to treatment plans are crucial for managing symptoms and improving quality of life. Healthcare providers can use the identified digital readiness profiles to tailor health interventions. For example, older adults or those with lower education levels may face more barriers to using digital tools. While these factors may influence the initial uptake of digital solutions, research suggests that they have less impact on outcomes once they are adopted (Hinman et al. 2024; Lawford et al. 2018; Nelligan, Hinman, McManus, et al. 2021). Furthermore, individuals who are initially hesitant due to low confidence in using digital tools often report positive experiences once they engage (Lawford et al. 2024). By recognising these dynamics, clinicians may tailor interventions, offering support or alternative in‐person solutions to ensure that all individuals, regardless of their digital readiness, may access health interventions.

The lack of research into digital readiness among individuals with hip or knee OA highlights the unique contribution of our study and underscores the need for further investigation. The identified digital readiness profiles can guide future studies in developing and validating tailored digital health interventions. Screening for digital readiness via instruments, such as the eHealth Readiness Scale, may help identify individuals (un)prepared to engage with digital solutions, addressing potential digital disparities (Rising et al. 2024). For comparative reasons, a clear definition and alignment of measurement instruments is a future research area, as existing studies use different methods to define and measure digital readiness, limiting direct comparisons (Rising et al. 2024; Yusif et al. 2017). Additionally, future research should employ longitudinal designs to understand how digital readiness evolves and what influences it, as well as identify effective enhancement strategies.

## 5 Conclusion

Our study revealed moderate digital readiness among individuals with hip or knee OA and low agreement on digital solution efficiency and utilization. We identified three digital readiness profiles distinguished by age, sex, education level, and number of comorbidities. This underscores the importance of considering digital readiness levels when designing and implementing digital health interventions to reduce digital disparities. Our results demonstrate the necessity of taking additional measures when introducing digital health solutions, emphasising that digital options should be one of several health intervention strategies. In‐person interventions remain essential for rehabilitation or exercise for those who are not ready to use digital health solutions. Therefore, the focus should be on a person‐centred approach, stratifying health initiatives to meet diverse needs and allocate healthcare more sustainably.

## Author Contributions

G.Z., L.H.T., L.T., D.G.T., E.M.R., and S.T.S. designed and conceptualised the study. G.Z., S.T.S., E.M.R., and D.G.T. contributed to data acquisition. G.Z. conducted the analysis and drafted the manuscript. S.T.S. provided senior supervision by interpreting findings and critically revising the manuscript. L.H.T., L.T., D.G.T., and E.M. contributed with substantive manuscript revisions. All authors reviewed and approved the final manuscript, agreed to be accountable for their contributions, and ensured the integrity and accuracy of the work.

## Ethics Statement

Ethical approval for GLA:D was waived by the North Denmark Region's ethics committee. The GLA:D registry is approved by the Danish Data Protection Agency (SDU; 10.084, 11.847). Under the Danish Data Protection Act, patient consent was not required as data were used for research and statistical purposes. This study adheres to the Helsinki Declaration.

## Conflicts of Interest

E.M.R. is the copyright holder of the KOOS and several other patient‐reported outcome measures and cofounders of GLA:D, a not‐for‐profit initiative to implement clinical guidelines in primary care hosted by the University of Southern Denmark. S.T.S. has received personal fees from Munksgaard, TrustMe‐Ed, and Nestlé Health Science outside the submitted work and is cofounder of GLA:D. All other authors declared no conflicts of interest.

## Supporting information

Supporting Information S1

## Data Availability

The data that support the findings of this study are available from the corresponding author upon reasonable request.
