# Supplementary material for: Digital Readiness Among 3555 Individuals With Hip or Knee Osteoarthritis Initiating a Supervised Education and Exercise Therapy Programme: A Cross‐Sectional Study
Source: Musculoskeletal Care. 2025 Jun 9;23(2):e70127. doi: 10.1002/msc.70127 (PMC12149361; doi:10.1002/msc.70127)
Supplement: Supplementary file 1 — Supporting Information S1 [file MSC-23-e70127-s001.pdf]

Supplementary Material

To the paper: **Digital Readiness among 3,555 Individuals with Hip or Knee Osteoarthritis Initiating a Supervised Education and Exercise Therapy Program – a cross-sectional study**

Supplementary Table 1. Reporting checklist for cross-sectional study following the Strengthening the Reporting of Observational Studies in Epidemiology (STROBE) guidelines

Supplementary Table 2. Description of the seven items in the eHealth Readiness Scale

Translation discrepancies and resolutions of the Danish version of the eHealth Readiness Scale

Supplementary Material Table 3. Variance inflation factors (VIF) for variables included in the multinomial logistic regression model

Suplementary Table 4. Nonresponders analysis

Supplementary material Table 5. Reliability of the eHealth Readiness scale

Supplementary material Table 6. Interitem correlations of the eHealth Readiness scale

Supplementary material Table 7. Latent class numbers and proportions

Supplementary material Table 8. Average latent lass probabilities for most likely latent class membership of model 1

Supplementary material Table 9. Average latent class probabilities for most likely latent class membership of model 2

Supplementary material Table 10. Average latent class probabilities for most likely latent class membership of model 3

Supplementary Table 11. Multinomial logistic regression of potential confounders associated with low vs. high digital readiness profiles

Supplementary Table 12. Multinomial logistic regression analysis of potential confounders associated with the intermediate digital readiness profile

Supplementary Table 13. Multinomial logistic regression analysis of potential confounders associated with the low digital readiness profile

Supplementary Table 14: Chi test of digital readiness profile and sex

Supplementary Table 15. Interaction analyses between age, sex, body mass index (BMI), and education in association with the low digital readiness profile vs high

Supplementary Table 16: Interaction analyses between age, sex, body mass index (BMI), and education in association with the intermediate digital readiness profile vs high

Supplementary Table 17: Interaction analyses between age, sex, body mass index (BMI), and education in association with the low digital readiness profile vs intermediate

Supplementary Table 1. Reporting checklist for cross-sectional study following the Strengthening the Reporting of Observational Studies in Epidemiology (STROBE) guidelines

Reporting checklist for cross sectional study.

		Reporting Item	Page Number
**Title and abstract**			
Title	#1a	Indicate the study's design with a commonly used term in the title or the abstract	1
Abstract	#1b	Provide in the abstract an informative and balanced summary of what was done and what was found	3
**Introduction**			
Background / rationale	#2	Explain the scientific background and rationale for the investigation being reported	5
Objectives	#3	State specific objectives, including any prespecified hypotheses	6
**Methods**			
Study design	#4	Present key elements of study design early in the paper	6
Setting	#5	Describe the setting, locations, and relevant dates, including periods of recruitment, exposure, follow-up, and data collection	6
Eligibility criteria	#6a	Give the eligibility criteria, and the sources and methods of selection of participants.	6
	#7	Clearly define all outcomes, exposures, predictors, potential confounders, and effect modifiers. Give diagnostic criteria, if applicable	7-10
Data sources / measurement	#8	For each variable of interest give sources of data and details of methods of assessment (measurement). Describe comparability of assessment methods if there is more than one group. Give information separately for for exposed and unexposed groups if applicable.	7-10

Bias	#9	Describe any efforts to address potential sources of bias	7-10
Study size	#10	Explain how the study size was arrived at	6-7
Quantitative variables	#11	Explain how quantitative variables were handled in the analyses. If applicable, describe which groupings were chosen, and why	7-10
Statistical methods	#12a	Describe all statistical methods, including those used to control for confounding	10-11
Statistical methods	#12b	Describe any methods used to examine subgroups and interactions	10-11
Statistical methods	#12c	Explain how missing data were addressed	10
Statistical methods	#12d	If applicable, describe analytical methods taking account of sampling strategy	NA
Statistical methods	#12e	Describe any sensitivity analyses	NA
**Results**			
Participants	#13a	Report numbers of individuals at each stage of study—eg numbers potentially eligible, examined for eligibility, confirmed eligible, included in the study, completing follow-up, and analysed. Give information separately for for exposed and unexposed groups if applicable.	12
Participants	#13b	Give reasons for non-participation at each stage	10,12
Participants	#13c	Consider use of a flow diagram	NA
Descriptive data	#14a	Give characteristics of study participants (eg demographic, clinical, social) and information on exposures and potential confounders. Give information separately for exposed and unexposed groups if applicable.	12, Table 1
Descriptive data	#14b	Indicate number of participants with missing data for each variable of interest	Table 1
Outcome data	#15	Report numbers of outcome events or summary measures. Give information separately for exposed and unexposed groups if applicable.	NA

Main results	#16a	Give unadjusted estimates and, if applicable, confounder-adjusted estimates and their precision (eg, 95% confidence interval). Make clear which confounders were adjusted for and why they were included	Suppl. Table 8-14
Main results	#16b	Report category boundaries when continuous variables were categorized	7-10
Main results	#16c	If relevant, consider translating estimates of relative risk into absolute risk for a meaningful time period	NA
Other analyses	#17	Report other analyses done—e.g., analyses of subgroups and interactions, and sensitivity analyses	10
**Discussion**			
Key results	#18	Summarise key results with reference to study objectives	14
Limitations	#19	Discuss limitations of the study, taking into account sources of potential bias or imprecision. Discuss both direction and magnitude of any potential bias.	18
Interpretation	#20	Give a cautious overall interpretation considering objectives, limitations, multiplicity of analyses, results from similar studies, and other relevant evidence.	14-19
Generalisability	#21	Discuss the generalisability (external validity) of the study results	18
**Other Information**			
Funding	#22	Give the source of funding and the role of the funders for the present study and, if applicable, for the original study on which the present article is based	2

The STROBE checklist is distributed under the terms of the Creative Commons Attribution License CC-BY. This checklist was completed on 29. September 2024 using https://www.goodreports.org/, a tool made by the EQUATOR Network in collaboration with Penelope.ai

Supplementary Table 2. Description of the seven items in the eHealth Readiness Scale

**Item 1**	I would be comfortable using an internet-connected device several times a week to participate in a lifestyle intervention	Jeg ville føle mig tryg ved at anvende en internetforbundet enhed flere gange om ugen for at deltage i en online livsstilsintervention
**Item 2**	I feel that my previous experiences with online technologies are important to my success with using a lifestyle intervention	Jeg føler at mine tidligere erfaringer med online teknologi har betydning for, om jeg opnår succes med at deltage i en online livsstilsintervention
**Item 3**	Using internet technologies makes me more efficient in my daily functioning	Anvendelse af internetteknologier gør, at jeg mere effektivt kan udføre dagligdagsfunktioner
**Item 4**	I believe that I am able to make good use of internet websites and web applications	Jeg synes jeg kan gøre god brug af internet hjemmesider og web programmer (apps)
**Item 5**	Using internet technologies provide me with a feeling of independence	Anvendelse af internetteknologier giver mig en følelse af uafhængighed
**Item 6**	I enjoy the challenge of figuring out the different functions of websites and web applications	Jeg kan godt lide udfordring ved finde ud af hvordan forskellige funktioner på internet hjemmesider og web programmer (apps) virker
**Item 7**	I use an internet connected device to keep track of my lifestyle (e.g., daily tasks, goals, and meetings)	Jeg anvender en internetforbundet enhed til at holde styr på min livsstil (fx dagens opgaver, mål og møder)

For the complete version of the eHealth readiness Scale, please contact Dr. Arjun Bhalla, see publication: Bhalla A, Durham RL, Al-Tabaa N, Yeager C. The development and initial psychometric validation of the eHealth readiness scale. Computers in Human Behavior. 2016 Dec 1;65:460–7.

Translation discrepancies and resolutions of the Danish version of the eHealth Readiness Scale

The main comment from the patient partners was if the introduction text was needed or if each item could be changed to incorporate this text to avoid sounding too academic. However, this was not accommodated to stay true to the original questionnaire. There were a few translation discrepancies and suggestions from the patient partners in items 1, 2, 4, and 6. One issue the Danish translator raised was using a Danish translation of the word ' web application' (used in the introduction text, items 4 and 6). This can be translated into Danish, but the original English word could also be used, so a compromise was chosen (i.e., *web programmer (apps)*). For item 1 (*I would be comfortable using an internet-connected device several times a week to participate in a lifestyle intervention online*), back translating meant that the item had more of an imperative 'having to use' instead of 'using' and the Danish wording was changed to reflect this. In item 2 (*I feel that my previous experiences with online technologies are important to my success using a lifestyle intervention online*), 'I feel' was left out in the forward translation but added in the final version to stay true to the original item. In the translation of item 6 (*I enjoy the challenge of figuring out the different functions of websites and web applications*), the English translator questioned whether the Danish translation implied more of a challenge instead of a more neutral tone, but the translation was kept as no other Danish word for 'challenge' was found more neutral. For the response categories, partly agree/disagree was chosen in Danish instead of mildly, as this is often used in Danish surveys.

Supplementary Material Table 3. Variance inflation factors (VIF) for variables included in the multinomial logistic regression model

Variable	VIF	1/VIF
**Age at first visit (per 10-year increase)**	1.43	0.700878
**Sex, male (ref. female)**	1.1	0.907564
**Born in Denmark (ref. yes)**	1.47	0.681018
**Danish citizenship (ref. yes)**	1.47	0.682579
Upper secondary education	2.97	0.33674
Higher education	3.08	0.325123
**Cohabitate status, living alone (ref. living with others)**	1.07	0.934033
Preobese	1.58	0.6337
Obese (class I, II, or III)	1.84	0.544396
**Self-reported most affected joint, hip (ref. knee)**	1.08	0.928508
**Bilateral symptoms (ref. no)**	1.09	0.915159
**Symptoms length of most affected joint in months**	1.03	0.972342
**Hip or knee pain during the last week (per 10-point increase)**	1.95	0.512517
**Taking pain medications (ref. no)**	1.2	0.836469
**Number of comorbidities**	1.2	0.836763
**Compliant with WHO's minimum recommendations for physical activity (ref. noncompliant)**	1.06	0.945701
**Nonsedentary behavior (ref. sedentary (sitting >9 h/day)**	1.03	0.96886
**KOOS 12/HOOS 12 summary score (per 10-point increase)**	2.21	0.452322
**EQ-5D VAS (per 10-point increase)**	1.37	0.730611
**Number of stands (30-second chair stand test)**	1.47	0.681858
**Walking speed meters per second (40-meter walk test)**	1.81	0.551904
**Mean VIF**	1.55	

Suplementary Table 4. Nonresponders analysis

Factor	Responders	Nonresponders
**Sample, n (%)**	3555	1221
**Age at first visit, mean (SD)**	66.4 (9.6)	67.3 (11.0)
**Sex, n (%)**		
Female	2386 (67.1%)	838 (68.6%)
Male	1169 (32.9%)	383 (31.4%)
**BMI, mean (SD)**	28.7 (5.5)	28.5 (5.6)
**BMI category (collapsed), n (%)**		
Underweight/normal weight	942 (26.5%)	341 (27.9%)
Preobese	1351 (38.0%)	456 (37.3%)
Obese (class I, II, or III)	1215 (34.2%)	394 (32.3%)
Missing	47 (1.3%)	30 (2.5%)
**Physio assessed most affected joint, n (%)**		
Knee	2361 (66.4%)	865 (70.8%)
Hip	1194 (33.6%)	356 (29.2%)
**Symptoms length of most affected joint in months, median (IQR)**	12.0 (6.0-27.0)	12.0 (5.0-24.0)
**Intake of pain medications, n (%)**		

No	1278 (35.9%)	449 (36.8%)
Yes	2277 (64.1%)	772 (63.2%)
**Number of stands (30 seconds chair stand test), mean (SD)**	11.9 (4.2)	11.0 (4.1)
**Walking speed meters per second (40 meters walk test), mean (SD)**	1.5 (0.4)	1.4 (0.4)
**Using walking aids during the 40 meters walk test, n (%)**		
Yes	55 (1.5%)	46 (3.8%)
No	3280 (92.3%)	1060 (86.8%)
Missing	220 (6.2%)	115 (9.4%)
**E-mail contact, n (%)**		
No	21 (0.6%)	106 (8.7%)
Yes	3534 (99.4%)	1115 (91.3%)

Supplementary material Table 5. Reliability of the eHealth Readiness scale

Item	Observations	Item-test correlation	Item-rest correlation	Average interitem covariance	Cronbach's alpha
**Item 1**	3,555	0.7828	0.6954	0.5654	0.8864
**Item 2**	3,555	0.7393	0.6385	0.5814	0.8929
**Item 3**	3,555	0.8356	0.7661	0.5459	0.8782
**Item 4**	3,555	0.8365	0.7674	0.5456	0.8781
**Item 5**	3,555	0.8497	0.7854	0.5407	0.8760
**Item 6**	3,555	0.7796	0.6911	0.5666	0.8869
**Item 7**	3,555	0.7105	0.6015	0.5921	0.8970
**Test scale**	3,555			0.5625	0.9000

Supplementary material Table 6. Interitem correlations of the eHealth Readiness scale

Items	Item 1	Item 2	Item 3	Item 4	Item 5	Item 6	Item 7
**Item 1**	1.0000						
**Item 2**	0.6083	1.0000					
**Item 3**	0.6289	0.6061	1.0000				
**Item 4**	0.5946	0.5087	0.6453	1.0000			
**Item 5**	0.5746	0.5313	0.6758	0.7381	1.0000		
**Item 6**	0.4904	0.4520	0.5549	0.6155	0.6564	1.0000	
**Item 7**	0.4353	0.3851	0.5133	0.5272	0.5260	0.5451	1.0000
Interitem correlations (reverse applied) (3,555 observations in all pairs)							

Supplementary material Table 7. Latent class numbers and proportions

Model and profile numbers	Latent Classes/profiles (higher class/profile number equals higher readiness)					Entropy
**Model 1 with 3 profiles (proportion)**	740 (0.2816)	1528 (0.42982)	1287 (0.36203)			0.873
**Model 2 with 4 profiles (proportion)**	310 (0.0872)	966 (0.27173)	1740 (0.48945)	539 (0.15162)		0.900
**Model 3 with 5 profiles (proportion)**	324 (0.09114)	498 (0.14008)	1175 (0.33052)	1094 (0.30774)	464 (0.13052)	0.880

Supplementary material Table 8. Average latent lass probabilities for most likely latent class membership of model 1

**Model 1 with 3 profiles**			
**Profiles**	**1**	**2**	**3**
**1**	0.958	0.027	0.004
**2**	0.038	0.935	0.038
**3**	0.004	0.053	0.943

Supplementary material Table 9. Average latent class probabilities for most likely latent class membership of model 2

**Model 2 with 4 profiles**				
**Profiles**	**1**	**2**	**3**	**4**
**1**	0.968	0.024	0.005	0.004
**2**	0.014	0.934	0.052	0.018
**3**	0.001	0.036	0.945	0.000
**4**	0.003	0.002	0.041	0.953

Supplementary material Table 10. Average latent class probabilities for most likely latent class membership of model 3

**Model 3 with 5 profiles**					
**Profiles**	**1**	**2**	**3**	**4**	**5**
**1**	0.955	0.022	0.016	0.002	0.004
**2**	0.009	0.933	0.050	0.008	0.001
**3**	0.003	0.031	0.908	0.057	0.001
**4**	0.01	0.003	0.057	0.913	0.026
**5**	0.001	0.001	0.004	0.001	0.944

Supplementary Table 11. Multinomial logistic regression of potential confounders associated with low vs. high digital readiness profiles

Model	Exposure Variable	Variables	Odds ratio (95% confidence interval)	% Change in odds ratio
**Adjusted model**	**Age (10-year)**	**All (see Table 2)**	**1.96 (1.71-2.24)**	-
Unadjusted model		Without sex, BMI, and educational level	1.90 (1.67-2.16)	3.1%
Model 1		Adding sex	1.95 (1.71-2.21)	-0.5%
Model 2		Adding BMI	1.85 (1.62-2.11)	-5.6%
Model 3		Adding educational level	1.95 (1.71-2.21)	-0.5%
**Adjusted model**	**Sex (male)**	**All (see Table 2)**	**0.72 (0.44-1.65)**	-
Unadjusted model		Without age (10-year), BMI, and educational level	0.92 (0.74-1.13)	27.8%
Model 1		Adding age (10-year),	0.76 (0.61-0.95)	5.6%
Model 2		Adding BMI	0.91 (0.74-1.13)	26.4%
Model 3		Adding educational level	0.87 (0.70-1.08)	20.8%
**Adjusted model**	**BMI (obesity)**	**All (see Table 2)**	**0.73 (0.54-0.97)**	-
Unadjusted model		Without age (10-year), sex, and educational level	0.53 (0.40-0.69)	27.4%
Model 1		Adding age (10-year)	0.74 (0.56-0.99)	1.4%
Model 2		Adding sex	0.53 (0.40-0.70)	27.4%
Model 3		Adding educational level	0.49 (0.37-0.65)	6.3%
**Adjusted model**	**Educational level (higher)**	**All (see Table 2)**	**0.32 (0.23-0.47)**	-
Unadjusted model		Without age (10-year), sex, and BMI	0.36 (0.25-0.50)	12.5%
Model 1		Adding age (10-year)	0.35 (0.25-0.49)	9.4%
Model 2		Adding sex	0.35 (0.25-0.49)	9.4%
Model 3		Adding BMI	0.34 (0.24-0.48)	6.3%
**Adjusted model**	**Sedentary behavior (sitting < 9 hours/day)**	**All (see Table 2)**	**1.63 (1.32-2.02)**	-
Unadjusted model		Without age (10-year), sex, education, and BMI	1.86 (1.51-2.28)	14.1%
Model 1		Adding age (10-year)	1.73 (1.40-2.13)	6.1%
Model 2		Adding sex	1.85 (1.51-2.28)	13.5%
Model 3		Adding BMI	1.78 (1.44-2.19)	9.2%
Model 4		Adding educational level	1.82 (1.48-2.24)	11.7%

Reference: high digital readiness profile; variables [reference] include age [per 10-year increase], sex [male], BMI; body mass index [obesity], education [higher level], and sedentary behavior [sitting >9 hours/day]

Supplementary Table 12. Multinomial logistic regression analysis of potential confounders associated with the intermediate digital readiness profile

Model	Exposure Variable	Variables	Odds ratio (95% confidence interval)	% Change in odds ratio
**Adjusted model**	**Age (10-year)**	**All (see Table 2)**	**1.30 (1.18-1.44)**	-
Unadjusted model		Without sex, BMI, and educational level	1.26 (1.15-1.39)	-3.1%
Model 1		Adding sex	1.31 (1.19-1.44)	0.8%
Model 2		Adding BMI	1.22 (1.11-1.35)	-6.2%
Model 3		Adding educational level	1.29 (1.17-1.42)	-0.8%
**Adjusted model**	**Sex (male)**	**All (see Table 2)**	**0.65 (0.55-0.78)**	-
Unadjusted model		Without age (10-year), BMI, and educational level	0.74 (0.63-0.88)	13.8%
Model 1		Adding age (10-year),	0.68 (0.58-0.81)	4.6%
Model 2		Adding BMI	0.74 (0.62-0.88)	13.8%
Model 3		Adding educational level	0.72 (0.61-0.85)	10.8%
**Adjusted model**	**BMI (obesity)**	**All (see Table 2)**	**0.75 (0.60-0.95)**	-
Unadjusted model		Without age (10-year), sex, and educational level	0.67 (0.54-0.83)	-10.7%
Model 1		Adding age (10-year),	0.75 (0.60-0.94)	0%
Model 2		Adding sex	0.68 (0.55-0.85)	-9.3%
Model 3		Adding educational level	0.64 (0.51-0.80)	14.7%
**Adjusted model**	**Educational level (higher)**	**All (see Table 2)**	**0.55 (0.40-0.75)**	-
Unadjusted model		Without age (10-year), sex, and educational level	0.59 (0.43-0.79)	7.3%
Model 1		Adding age (10-year),	0.58 (0.43-0.79)	5.5%
Model 2		Adding sex	0.58 (0.42-0.77)	5.5%
Model 3		Adding BMI	0.57 (0.42-0.78)	3.6%
**Adjusted model**	**Sedentary behavior (sitting <9 hours/day)**	**All (see Table 2)**	**1.20 (1.02-1.42)**	-
Unadjusted model		Without age (10-year), sex, education, and BMI	1.33 (1.13-1.56)	10.8%
Model 1		Adding age (10-year)	1.28 (1.09-1.50)	6.7%
Model 2		Adding sex	1.31 (1.12-1.54)	9.2%
Model 3		Adding BMI	1.28 (1.09-1.50)	6.7%
Model 4		Adding educational level	1.32 (1.12-1.55)	10.0%

Reference: high digital readiness profile; variables [reference] include age [per 10-year increase], sex [male], BMI; body mass index [obesity], education [higher level], and sedentary behavior [sitting >9 hours/day]

Supplementary Table 13. Multinomial logistic regression analysis of potential confounders associated with the low digital readiness profile

Model	Exposure Variable	Variables	Odds ratio (95% confidence interval)	% Change in odds ratio
**Adjusted model**	**Age (10-year)**	**All (see Table 2)**	**1.50 (1.32-1.71)**	-
Unadjusted model		Without sex, BMI, and educational level	1.50 (133-1.70)	0%
Model 1		Adding Sex	1.49 (1.32-1.68)	-0.7%
Model 2		Adding BMI	1.51 (1.33-171)	0.7%
Model 3		Adding, educational level	1.22 (0.99-1.50)	18.7%
**Adjusted model**	**Sex (male)**	**All (see Table 2)**	**1.10 (0.89-1.36)**	-
Unadjusted model		Without age (10-year), BMI, and educational level	1.23 (1.00-1.52)	11.8%
Model 1		Adding age (10-year),	1.11 (0.90-1.38)	0.9%
Model 2		Adding BMI	1.23 (1.00-1.52)	11.8%
Model 3		Adding educational level	1.22 (0.99-1.50)	11.8%
**Adjusted model**	**BMI (obesity)**	**All (see Table 2)**	**0.96 (0.73-1.26)**	-
Unadjusted model		Without age (10-year), sex, and educational level	0.79 (0.61-1.01	17.7%
Model 1		Adding age (10-year),	0.99 (0.76-1.29)	3.1%
Model 2		Adding sex	0.78 (0.60-1.00)	18.8%
Model 3		Adding educational level	0.77 (0.60-1.00)	20.8%
**Adjusted model**	**Educational level (higher)**	**All (see Table 2)**	**0.60 (0.44-0.81)**	-
Unadjusted model		Without age (10-year), sex, and educational level	0.61 (0.45-0.82)	1.7%
Model 1		Adding age (10-year),	0.60 (0.44-0.81)	0%
Model 2		Adding sex	0.62 (0.46-0.83)	3.3%
Model 3		Adding BMI	0.60 (0.44-0.81)	0%

Reference: intermediate digital readiness profile; variables [reference] include age [per 10-year increase], sex [male], BMI; body mass index [obesity], education [higher level], and sedentary behavior [sitting >9 hours/day]

Supplementary Table 14: Chi test of digital readiness profile and sex

Digital readiness profile	Female	Male	Total	Pearson chi2(2)
**Low digital readiness**	514 (69.5%)	226 (30.5%)	740 (100.0%)	
**Intermediate digital readiness**	1083 (70.9%)	445 (29.1)	1528 (100.0%)	
**High digital readiness**	789 (61.3%)	498 (38.7%)	1287 (100.0%)	
**Total**	2386 (67.1%)	1169 (32.9%)	3555 (100.0%)	p=<0.001

Supplementary Table 15. Interaction analyses between age, sex, body mass index (BMI), and education in association with the low digital readiness profile vs high

Interaction terms	Odds ratio (95% confidence interval)	p-value
Age (per 10-year)*	2.03 (1.74-2.38)	<0.001
Sex (male)	1.52 (0.27-8.66)	0.640
Sex (male)#age (per 10-year)	0.89 (0.70-1.15)	0.384
Age (per 10-year)*	2.12 (1.66-2.71)	<0.001
BMI (preobese)	1.04 (0.12-9.00)	0.972
BMI (obese)	2.63 (0.32-21.81)	0.371
BMI (preobese)#age (per 10-year)	0.97 (0.71-1.32)	0.849
BMI (obese)#age (per 10-year)	0.83 (0.61-1.13)	0.232
Age (per 10-year)	1.78 (0.83-1.67	0.363
Educational level (upper secondary)*	0.02 (0.00-0.27)	0.004
Educational level (higher)*	0.00 (0.00-0.06)	<0.001
Educational level (upper secondary)#age (per 10-year)*	1.65 (1.13-2.42)	0.010
Educational level (higher)#age (per 10-year)*	1.91 (1.28-2.84)	0.001
Sex (male)	0.84 (0.55-1.28)	0.420
BMI (preobese)	0.96 (0.70-1.32)	0.804
BMI (obese)	0.72 (0.51-1.01)	0.056
BMI (preobese)#sex (male)	0.67 (0.39-1.15)	0.145
BMI (obese)#sex (male)	1.02 (0.58-1.79)	0.945

Reference: High Readiness Profile

BMI; Body mass index

* indicates significant interaction

Supplementary Table 16: Interaction analyses between age, sex, body mass index (BMI), and education in association with the intermediate digital readiness profile vs high

Interaction terms	Odds ratio (95% confidence interval)	p-value
Age (per 10-year)*	1.35 (1.20-1.52)	<0.001
Sex (male)	1.41 (0.41-4.87)	0.584
Sex (male)#age (per 10-year)	0.89 (0.74-1.07)	0.215
Age (per 10-year)*	1.29 (1.08-1.54)	0.005
BMI (preobese)	0.84 (0.19-3.74)	0.817
BMI (obese)	0.65 (0.15-2.87)	0.566
BMI (preobese)#age (per 10-year)	1.01 (0.81-1.26)	0.928
BMI (obese)#age (per 10-year)	1.02 (0.82-1.28)	0.843
Age (per 10-year)	0.87 (0.63-1.19)	0.378
Educational level (upper secondary)*	0.08 (0.01-0.87)	0.038
Educational level (higher)*	0.01 (0.00-0.12)	<0.001
Educational level (upper secondary)#age (per 10-year)	1.40 (1.00-1096)	0.051
Educational level (higher)#age (per 10-year)*	1.77 (1.26-2.50)	0.001
BMI (preobese)	0.97 (0.44-2.14)	0.942
BMI (obese)	0.99 (0.44-2.21)	0.975
Educational level (upper secondary)	0.96 (0.47-1.33)	0.917
Educational level (higher)	0.66 (0.32-1.33)	0.242
BMI (preobese)#educational level (upper secondary)	0.90 (0.39-2.18)	0.817
BMI (preobese)#educational level (higher)	0.94 (0.41-2.18)	0.888
BMI (obese)#educational level (upper secondary)	0.83 (0.35-1.97)	0.674
BMI (obese)#educational level (higher)	0.66 (0.28-1.56)	0.341

Reference: High Readiness Profile

BMI; Body mass index

* indicates significant interaction

Supplementary Table 17: Interaction analyses between age, sex, body mass index (BMI), and education in association with the low digital readiness profile vs intermediate

Interaction terms	Odds ratio (95% confidence interval)	p-value
Age (per 10-year)*	1.50 (1.30-1.74)	<0.001
Sex (male)	1.07 (0.20-5.87	0.936
Sex (male)#age (per 10-year)	1.01 (0.79-1.28)	0.964
Age (per 10-year)*	1.64 (1.31-2.06)	<0.001
BMI (pre-obese)	1.24 (0.16-9.59)	0.837
BMI (obese)	4.07 (0.54-30.49)	0.172
BMI (pre-obese)#age (per 10-year)	0.96 (0.72-1.28)	0.784
BMI (obese)#age (per 10-year)	0.81 (0.61-1.08)	0.153
Age (per 10-year)*	1.36 (1.01-1.82)	0.042
Educational level (upper secondary)	0.22 (0.02-2.23)	0.203
Educational level (higher)	0.34 (0.03-3.95)	0.388
Educational level (upper secondary)#age (per 10-year)	1.18 (0.85-1.63)	0.317
Educational level (higher)#age (per 10-year)	1.08 (0.76-1.52)	0.676

Reference: Intermediate Readiness Profile

BMI; Body mass index

* indicates significant interaction
